# Cardiac cell senescence: molecular mechanisms, key proteins and therapeutic targets

**DOI:** 10.1038/s41420-023-01792-5

**Published:** 2024-02-14

**Authors:** Yi Luan, Xiaofan Zhu, Yuxue Jiao, Hui Liu, Zhen Huang, Jinyan Pei, Yawei Xu, Yang Yang, Kaidi Ren

**Affiliations:** 1 Clinical Systems Biology Laboratories, The First Affiliated Hospital of Zhengzhou University, Zhengzhou, 450052 P. R. China; 2 Genetic and Prenatal Diagnosis Center, Department of Obstetrics and Gynecology, the First Affiliated Hospital of Zhengzhou University, Zhengzhou, 450052 P. R. China; 3 School of Laboratory Medicine, Xinxiang Medical University, Xinxiang, 453003 P. R. China; 4 Quality Management Department, Henan No.3 Provincial People’s Hospital, Zhengzhou, 450052 P. R. China; 5 Department of Cardiovascular Medicine, The First Affiliated Hospital of Zhengzhou University, Zhengzhou, 450052 P. R. China; 6 Department of Pharmacy, the First Affiliated Hospital of Zhengzhou University, Zhengzhou, 450052 P. R. China; 7 Henan Key Laboratory of Precision Clinical Pharmacy, Zhengzhou University, Zhengzhou, 450052 P. R. China

**Keywords:** Heart failure, Senescence

## Abstract

Cardiac aging, particularly cardiac cell senescence, is a natural process that occurs as we age. Heart function gradually declines in old age, leading to continuous heart failure, even in people without a prior history of heart disease. To address this issue and improve cardiac cell function, it is crucial to investigate the molecular mechanisms underlying cardiac senescence. This review summarizes the main mechanisms and key proteins involved in cardiac cell senescence. This review further discusses the molecular modulators of cellular senescence in aging hearts. Furthermore, the discussion will encompass comprehensive descriptions of the key drugs, modes of action and potential targets for intervention in cardiac senescence. By offering a fresh perspective and comprehensive insights into the molecular mechanisms of cardiac senescence, this review seeks to provide a fresh perspective and important theoretical foundations for the development of drugs targeting this condition.

## Facts

- This review summarizes the main mechanisms and key proteins involved in cardiac cell senescence.
- This review discusses the molecular modulators of cellular senescence in aging hearts.
- This review seeks to provide a fresh perspective and important theoretical foundations for the development of drugs targeting this condition.

## Open questions

- What are the molecular modulators regulating cellular senescence in aging hearts?
- What are the main mechanisms involved in cardiac cell senescence?
- What are the potential targets for intervening in cardiomyocyte senescence?

## Introduction

According to data from the World Health Organization, cardiovascular diseases (CVDs) account for a mortality of 17.9 million every year worldwide, accounting for ~31% of all deaths [1]. Senescence is a major risk factor for many life-threatening disorders, including cardiovascular diseases, such as heart failure (HF). For individuals over 50 years old, ~1% of people suffer from HF, which will be twice as much with each decade of life. In the United States, the number of aged people (over 65 years old) increased from 40 million to 51 million in a decade and is estimated to reach 95 million in 2060. Given the dramatic increase in the aged population, age-related cardiovascular diseases have become one of the greatest challenges for global health care today. Meanwhile, heart diseases may make people have a poor quality of life, including various physical and emotional symptoms, such as dyspnea, fatigue, edema, difficulty sleeping, depression, and chest pain [2]. Poor life quality is also associated with high hospitalization and mortality rates.

The heart is a complex organ comprising four chambers with distinct morphologies and functions. Heart function generally declines with increasing age despite no prior history of heart disease, as the heart is in continuous failure. [3]. During senescence, cardiomyocytes develop a senescent-like phenotype, which finally leads to age-related myocardial dysfunction [4]. The aging myocardium acquires deleterious structural changes, including progressive cardiomyocyte hypertrophy, interstitial fibrosis, and inflammation, ultimately contributing to diastolic and systolic dysfunction. A more important part of the increased HF during aging can be attributed to a greater opportunity to be exposed to various stimuli, such as metabolic stress, hypertension, or ischemic injury [5]. The repair and regeneration capacity of the heart is limited, implying that old individuals have a greater tendency to develop cardiac disorders [6]. Given the increasing aging population, it is imperative to explore the molecular mechanisms of cardiac senescence for better intervention in senescence and enhancement of cardiac function.

### Cardiac senescence

Senescence is the gradual deterioration of bodily function that can occur at either the cellular or organismal level. Cellular senescence, or replicative senescence, is characterized by halted mitosis of diploid cells. Meanwhile, organismal senescence is characterized by a reduced stress response and increased homeostatic imbalance, resulting in a loss of normal functions in organisms [7].

Cellular senescence is the cellular basis of organismal aging and is a reliable model for studying organismal aging. It is defined as the arrest of cell cycle progression caused by telomere shortening or stress-induced premature senescence. The condition is characterized by flattened and enlarged cell morphology and lysosomal activation, resulting in positive staining for senescence-associated β-galactosidase activity (SA-β-gal) [8]. Additionally, stress can induce premature senescence and activate many markers similar to replicative senescence. Various experimental stressors, including oxidative stressors such as hydrogen peroxide, have been used for quick labeling of cellular senescence [9].

Cardiac senescence is generally considered a normal phenomenon. The possible causes include load or chronic myocardial lesions, such as chronic cardiac insufficiency, cardiac overload caused by coronary heart disease and hypertension, myocardial ischemia, hypertrophic heart disease, and pericardial disease [10].

### Cardiac cell type and function

**Fig. 1:**
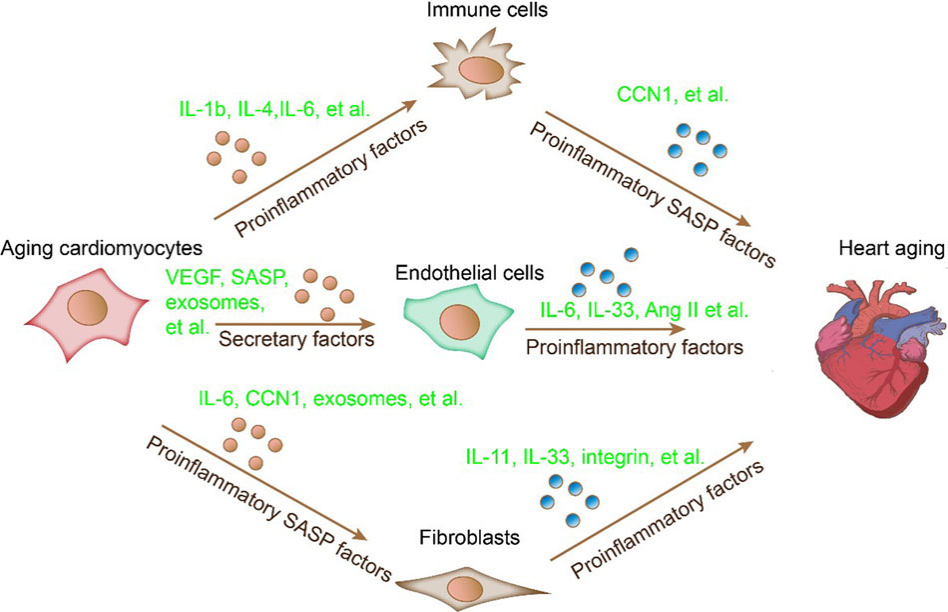
Cardiomyocytes and cardiac nonmyocytes interact with each other within the local microenvironment. Cardiac nonmyocytes modulate cardiomyocytes by releasing signals, leading to cardiac senescence. Dysfunctional endothelial cells (ECs) release proinflammatory factors (IL-6 and IL-33), Ang II, and ET-1 to promote cardiomyocyte senescence. Fibroblasts induce senescence by producing IL-11, IL-33, and integrin. Immune cells release various signals to regulate cardiomyocyte senescence directly. Reciprocally, senescent dysfunctional cardiomyocytes undergo a senescence-associated secretory phenotype (SASP) to recruit immune cells. Aging cardiomyocytes produce SASP, VEGF, and exosomes to induce senescence in ECs. Similarly, the functional impairment in fibroblasts is regulated by dysfunctional cardiomyocytes through the secretion of IL-6, CCN1, and exosomes.

Cardiac tissue is composed of various cell types, and the cooperation of distinct cell types contributes to the crucial functions of the heart. Previous pioneering research has revealed that the heart comprises 70% noncardiomyocytes and 30% cardiomyocytes, which can be grouped into atrial and ventricular myocytes [11]. Noncardiomyocytes include fibroblasts, smooth muscle cells (SMCs), pericytes, and endothelial cells (ECs) (Fig. 1). These cells constitute four chambers and function in systemic blood circulation. Cardiomyocytes are involved in contractile function and act in parallel with other cell types [12]. Fibroblasts account for >40% of the total cells in the ventricle. They mainly serve as mechanical and structural support for cardiomyocytes and sustain the homeostasis of the cardiac extracellular matrix [13]. Mural cells in the vessel wall include smooth muscle cells and pericytes, which are vital for vascular integrity and cardiac function [14]. ECs constitute the inner layer of blood and lymphatic vessels and are responsible for regulating blood circulation by changing the permeability and caliber of blood vessels [15]. In addition, ECs regulate and maintain heart growth, contractility, and rhythm [16]. As transitional mesodermal-derived cells, mesothelial cells share morphological and functional similarities with endothelial cells. They can produce angiogenic factors necessary for angiogenesis [17]. Meanwhile, adipose cells in the heart provide energy and repair heart tissues, including new blood vessel formation and immune modulation. Immune cells and neurons play vital roles in maintaining functional homeostasis.

### Cardiac cell senescence

At the cellular level, different types of cellular senescence are the basis of cardiac senescence. The replicative capacity of cells is limited, indicating that aging occurs at the cellular level, namely, as “cellular senescence”. Senescent cells exhibit cell cycle arrest, which initiates tissue remodeling, leading to development and injury responses. Senescence also leads to a decline in regenerative capacity and tissue function, including inflammation. The following discussion will introduce the effects of senescence on cardiomyocytes and cardiac nonmyocytes.

#### Senescence in cardiomyocytes

**Fig. 2:**
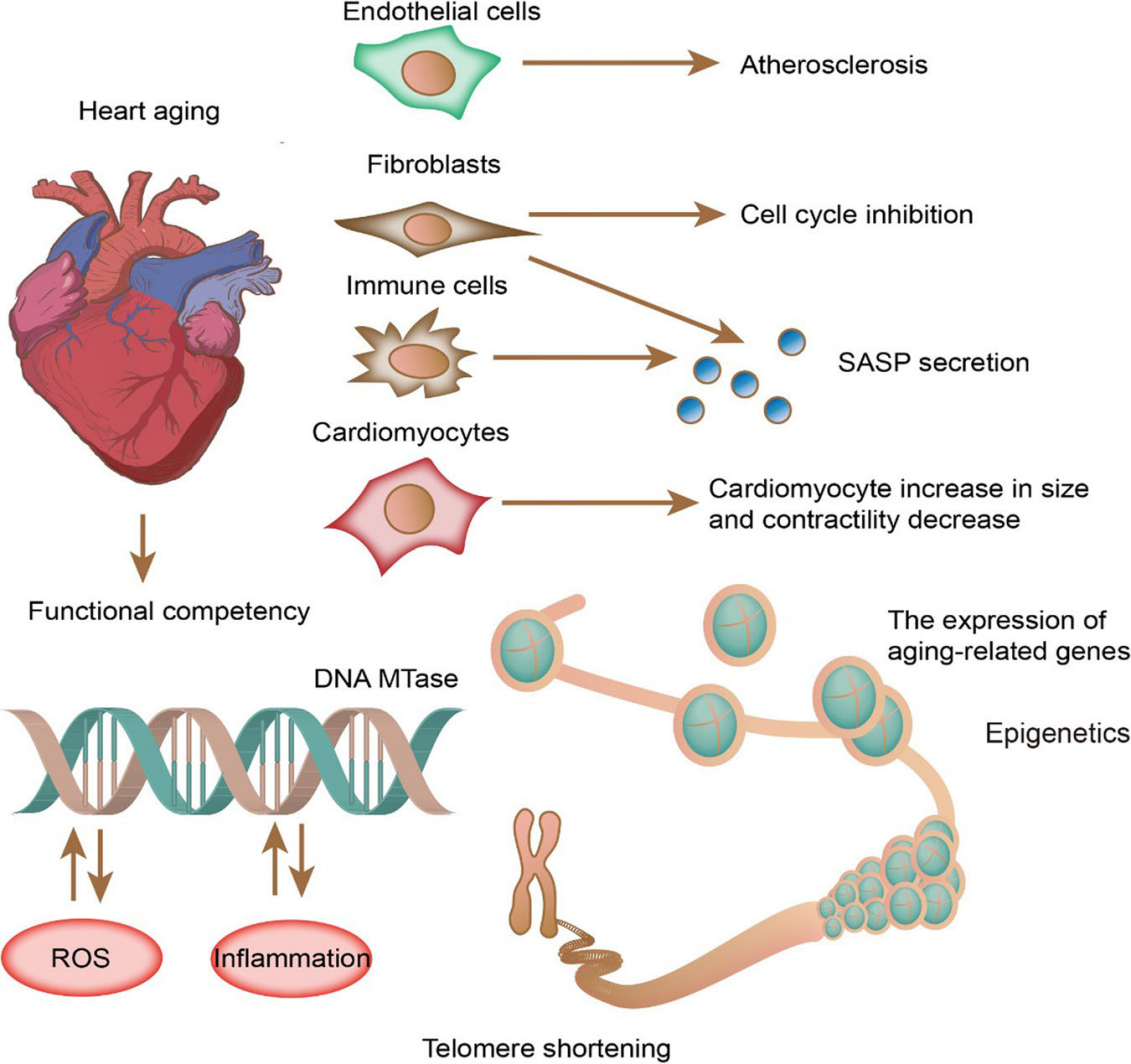
Cellular and molecular signals affect cardiac senescence. Cardiac cell dysfunction occurs in a cell- and tissue-specific manner: EC senescence is associated with atherosclerosis, and fibroblasts show cell cycle inhibition and SASP. Immune cells secrete SASP factors. Cardiomyocytes increase in size and lose contractility. At the molecular level, ROS and inflammation induce DNA damage and telomere shortening, leading to the expression of aging-related genes and SASP.

Cardiomyocytes account for ~80% of the cellular volume and 30%-40% of the total cell population in the heart. Thus, cardiomyocyte senescence affects normal heart function. Senescent cardiomyocytes are characterized by irregular shortening, elevated pacing frequency, dysregulated contraction, and SASP factor production, affecting neighboring cells, including fibroblasts [4]. Replicative senescence is typically triggered by the cell cycle inhibitory tumor-suppressor pathways p53-p21 (CDKN1A) and p16INK4A-retinoblastoma-associated protein [18]. Nonproliferative cardiomyocytes can be induced by inflammation and reactive oxygen species, leading to telomere shortening, DNA damage, functional damage, alterations in ploidy, and other molecular senescence signatures (Fig. 2) [19]. For instance, SASP is a typical senescence signature that includes the secretion of proinflammatory cytokines, chemokines, growth factors, and proteases [20]. Senescence-associated paracrine signaling maintains cell senescence or induces tissue healing depending on the tissue environment [21]. Senescent cardiomyocytes display functional decline, including reduced contractility, mitochondrial dysfunction, enlarged size, and shortened telomeres, mainly affecting the normal function of myocardia [5]. These poorly functional cardiomyocytes accumulate with age, block intracellular communication, and trigger chronic inflammation, cell death, and cardiomyocyte shortage [4]. Interestingly, inflammation, rather than telomere shortening, has been identified as the primary determinant of aging in Japanese centenarians [22]. At the cardiac level, coping with senescent cardiomyocytes has become a central approach for alleviating the adverse effects of aging on myocardial structure and function.

Metabolic stress has been identified as a cause of cardiomyocyte senescence. Metabolic disorders directly affect age-related functional disability in the heart because they can cause a shortage of energy supply, leading to cardiac senescence [4, 23]. Defects in mitochondrial dynamics and quality can accelerate ROS production and oxidative damage. Additionally, improper regulation of mitophagy may lead to cellular senescence owing to inefficiency in removing damaged mitochondria [24]. Cardiac-specific triple deletion of Drp1 and MFN1/2 prolonged the survival of mice compared to mice with deletion of either Drp1 or MFN1/2, indicating adverse effects of fusion and fission imbalance [25]. FOXO3 activation prevents oxidative damage and cardiomyocyte senescence [26]. Moreover, mitochondrial catalase overexpression prevents age-induced metabolic dysfunctions in the heart [27]. Accumulated mtDNA mutations during aging have been associated with elevated ROS production, activation of Toll-like receptor 9 (TLR9) signaling, and the release of proinflammatory cytokines, ultimately inducing an inflammatory response in cardiomyocytes [28]. These inflammatory responses maintain SASP phenotypes, promoting the progression of cardiovascular diseases. The findings imply that ROS are vital in inducing senescence in the heart (Fig. 2). Moreover, studies have also identified cell cycle regulators involved in senescence [7, 29]. Cytosolic p53 induces mitochondrial dysfunction by inhibiting Parkin-mediated mitophagy [30]. p53 activation induced by senescence can abrogate glucose metabolism in cardiomyocytes. Conversely, its inhibition protects against senescence and cardiomyopathy in diabetic mice [31]. Studies have revealed that the cell cycle inhibitors Rb1 and Meis homeobox 2 (Meis2) and epigenetic factors can also promote senescence [32]. Histone demethylase (KDM4D) overexpression promotes the expression of proliferative and cell cycle genes, inhibiting cell cycle arrest [33]. Aging hearts also show elevated miR-34 levels, reducing cell contractility [34]. These findings demonstrate the alleviation of cardiac hypertrophy, apoptosis, and senescence by modest SIRT1 overexpression [35, 36]. Similarly, SIRT7 protects against apoptosis and exerts antiaging effects in cardiomyocytes by interacting with and deacetylating p53 [37]. Senescent cardiomyocytes secrete the inflammatory cytokines IL-1/6, TNF-α, endothelin 3 (Edn3), growth and differentiation factor 15 (GDF15), and TGF-β. IL-1, IL-6, and TNF-α induce inflammation in the neighboring cardiac microenvironment. They also secrete Edn3, GDF-15, and TNF-β, resulting in fibrosis and myofibroblast activation in cardiac fibroblasts [38]. In general, SASP in cardiomyocytes is closely associated with surrounding cells via paracrine effects.

#### Senescence in endothelial cells

Endothelial cells (ECs) account for ~60% of noncardiomyocytes in the heart. ECs regulate vasodilation and vascular tone by producing vasoactive compounds and growth factors [39]. Senescence in ECs is associated with various diseases, such as atherosclerotic plaques, heart failure, and atrial fibrillation [40]. Oxidative stress or vascular inflammation evokes senescence in ECs [41] in addition to metabolic cues, such as hyperuricemia or a dysregulated renin-angiotensin system [42]. Epigenetic factors, such as miRNAs or acetylation, are also involved in EC senescence. A study reported aggravation of atherosclerosis in ApoE-deficient mice by miR-217-induced EC dysfunction [43]. Additionally, SIRT1 represses the replicative senescence pathway by deacetylating p53 in ECs. Enhanced endothelin-1 (ET-1) production and decreased nitric oxide levels in senescent ECs establish extensive crosstalk between senescent ECs and surrounding cardiac cells [44], ultimately inducing vascular inflammation and dysfunctional vasodilation. Therefore, senescent ECs have become a promising therapeutic target for the prevention and treatment of aging-related vascular dysfunction. These findings demonstrate effective alleviation of EC senescence by the neuregulin-1 (NRG1) effect via ErbB tyrosine kinase receptors [45]. Telomerase expression also relieves aging-related vascular dysfunction. The mediation of EC senescence provides a viable opportunity to prevent the downstream effects of senescence in other cardiac cell populations.

EC senescence may lead to atherosclerosis, heart failure, or pulmonary hypertension. SIRT6 depletion or miR-217 overexpression is associated with EC senescence, resulting in atherosclerosis [46]. Senescence-accelerated mice (SAMP8/TaHsd mice) exhibit metabolic disorders, including diastolic dysfunction, left ventricular hypertrophy, and interstitial fibrosis. The prevalence of atrial fibrillation increases with age, increasing morbidity and mortality associated with thromboembolic events and heart failure. Similarly, atrial fibrillation is associated with EC and fibroblast senescence caused by eNOS downregulation and EC dysfunction [47]. Findings have demonstrated a positive correlation between senescence markers (p53 and p16) and the severity of atrial fibrillation.

#### Senescence in cardiac fibroblasts

Cardiac fibroblasts account for 20% of the noncardiomyocyte population and are associated with ECM remodeling and paracrine communication in the cardiac microenvironment. Although fibroblast senescence can benefit chronic wound healing during myocardial infarction (MI), it can also aggravate myocardial fibrosis during aging. Cardiac fibroblasts retain their role in ECM adhesion by expressing integrins and MMPs. Acute MI promotes upregulation of p53/p21 pathways and cardiac fibroblast senescence. Cell communication network factor 1 (CCN1), a SASP protein, ameliorates myocardial fibrosis, possibly by inducing fibroblast senescence after MI. This finding suggests the potential benefits of fibroblast senescence under certain circumstances [48]. In contrast, NEIL3, a DNA glycosylase that effectively removes oxidatively damaged DNA, promotes cardiac rupture by enhancing MMP2 expression involved in ECM degradation [49]. Cardiac fibroblasts are also involved in proliferation, hypertrophic growth, and cardiomyocyte senescence by interfering with paracrine signaling. Heart injury induces the secretion of IL-1, IL-6, TNF-α, and TGF-β in cardiac fibroblasts. Cardiac fibroblasts also secrete insulin-like growth factor-1 (IGF-1) to promote collagen synthesis and cardiomyocyte hypertrophy [50]. Aging worsens inflammation, collagen deposition, fibrosis, myocardial stiffness, calcium turbulence, arrhythmias, and irregular cardiac function [51]. Moreover, senescent fibroblasts lead to deleterious remodeling in aging hearts.

#### Senescence in vascular smooth muscle cells

VSMCs function cooperatively with ECs to regulate blood pressure, vascular tone, and blood flow. Vascular and interstitial cardiac cells exhibit cell cycle arrest, inflammation activation, p16INK4A expression, telomere shortening, and acquisition of SASP during senescence [22]. Meanwhile, the accumulation of senescent cells and calcification are features of atherosclerotic plaques in the vasculature (Fig. 2) [52]. Telomere shortening, oxidative stress, DNA damage, and autophagic deficiency are among the factors known to induce senescence in VCMCs [53]. Senescent VSMCs show increased SA-β-Gal activity and p16/p21/Rb levels. Abnormal cleavage of prelamin A induces the incorrect accumulation of nuclear lamina, rendering cells vulnerable to DNA damage. Mutations in LMNA (Lamin A/C) result in elevated prelamin A, which contributes to VSMC senescence by reducing the expression of ZMPSTE, a metalloprotease that cleaves prelamin A. Accumulation of prelamin A leads to sustained DNA damage signaling and promotes the secretion of pro-osteogenic cytokines, leading to VSMC mineralization and vascular calcification [54]. Additionally, SIRT6 deficiency results in VSMC senescence due to hyperacetylation at H3K9 and H3K27, ultimately causing DNA telomere damage. Increased IL-6 and C-C motif chemokine ligand 2 (CCL2) levels were also reported in senescent VSMCs. SASP in senescent VSMCs also includes monocyte chemoattractant protein 1 (MCP1), CCL3/4, and IL-1/IL-6/IL-8, as well as reduced anti-inflammatory cytokines [53]. IL-1α expression activates SASP and aggravates IL-6 levels in surrounding cells, indicating a paracrine effect of senescent VSMCs on the local inflammatory microenvironment.

Similar to ECs, VSMC senescence also participates in blood vessel disorders, such as atherosclerosis and pulmonary hypertension. Plaque VSMCs show relatively short telomeres, elevated p16 and p21 levels, increased SA-β-gal activity, and enhanced oxidative stress compared to normal VSMCs [55]. Plaque VSMCs also demonstrated reduced SIRT6 and SIRT6 overexpression levels, relieving senescence in VSMCs. As observed in Hutchinson-Gilford progeria symptoms, accumulation of prelamin A in VSMCs induces premature symptoms. Patients with severe atherosclerosis are vulnerable to accelerated aging and premature diseases [56]. Furthermore, VSMC senescence is linked to the pathogenesis of pulmonary hypertension.

#### Senescence in valve interstitial cells

Valve interstitial cells (VICs) constitute the vast majority of cells in heart valves, and their senescence might disrupt valve function. The prevalence of aortic stenosis due to aortic valve leaflet calcification increases with age. Calcified aortic valves were previously associated with increased expression of p16 and p53, indicating the relevance of a senescent phenotype as a biomarker for aging [57]. Proinflammatory macrophages secrete IL-6, TNF-, and IL-1, eventually transforming into a transient osteogenic phenotype and promoting calcification and fibrosis in VICs. Similarly, AMPK activation induces senescence in porcine aortic VICs, accompanied by elevated ROS and calcified nodules [58]. Calcified VICs also exhibit elevated levels of hyperacetylation in histone 3, reduced miR-17 and miR-30a, and enhanced levels of p21 and SA-β-Gal activity.

### Alterations in cardiac cell senescence

#### Signaling pathway changes associated with senescence

The process of cardiac senescence is inseparable from a series of pathological alterations. Aging is prominently characterized by elevated and sustained endogenous levels of stress response signaling pathways, including p38 MAPK, SAPK/JNK and NF-κB [59]. These pathways are closely related to the initiation and promotion of senescence and CVD phenotypes. In age-associated chronic stress, alterations in the signaling network are indicators of inflammatory processes that induce enhanced endogenous inflammation and oxidative stress [59]. These signaling processes are critical factors in promoting the progression of stress-induced aging and tissue functional decline during aging. These characteristics also play an important role in the progression of aging-related oxidative stress. A study revealed that mitochondrial-derived ROS was presumably linked to the activation of stress-induced aging phenotypes, and ROS-responsive ASK1-sigalosomes modulated p38 MAPK pathway activation and subsequent senescence and aging [60]. This study implies that disease-induced oxidative stress is associated with the ASK1-p38 MAPK pathway. In fact, mitochondrial dysfunction is responsible for 90% of age-associated ROS production, which subsequently activates the p38 MAPK and SAPK/JNK pathways, indicating that these signaling pathways induce senescence, aging and oxidative stress diseases.

#### Epigenetic modification changes associated with senescence

In addition to signaling pathways, epigenetic alterations are also crucial for cardiac senescence. As indicated by limited studies, cardiac aging in murine and human models revealed a strong correlation between the epigenome, age and senescence [61]. Epigenetic modifications, such as DNA methylation, histone modifications, chromatin remodeling, and noncoding RNAs, display a unique profile in senescent cells. Senescent cells show a unique signature of DNA hypomethylation in late-replicative genes and certain promoter-proximal regions [62]. The impact of senescence on epigenetic modification and the potential regulatory role of epigenetic modification on cardiac senescence are discussed in detail in the following sections [61].

#### Proteomic/metabolomic changes associated with senescence

Proteomic analysis displayed mitochondrial adaptations in senescent cells. In a human fibroblast senescent model, broad reshaping of the mitochondrial proteome and metabolic rewiring were observed [63]. Senescence induced ~40% of mitochondrial protein changes accompanied by more membrane protein enrichment over soluble matrix proteins. Meanwhile, senescent cells showed increased branched-chain amino acid degradation and lipid metabolism, as well as reduced 1C-folate metabolism and OXPHOS activities associated with reduced protein translation [64]. Given that mitochondria govern the SASP profile, it is plausible that metabolic rewiring of mitochondria is essential for shaping the SASP and may modulate the impacts of senescent cells in the context of aging-related diseases [65].

The metabolomic profiles change significantly following the induction of senescence. Senescent cells showed increased free cytosolic polyunsaturated fatty acids, which are subsequently transformed to oxylipins, part of the SASP [64]. Senescence also induces the accumulation of lipid droplets. Sterol accumulates preferentially in the ER in a p53-dependent manner, limiting the activation of SREBP2. Moreover, senescent cells have increased mitochondrial mass or mtDNA, coupled with changed membrane potential and ROS generation [66].

### Molecular modulators in cell senescence

**Fig. 3:**
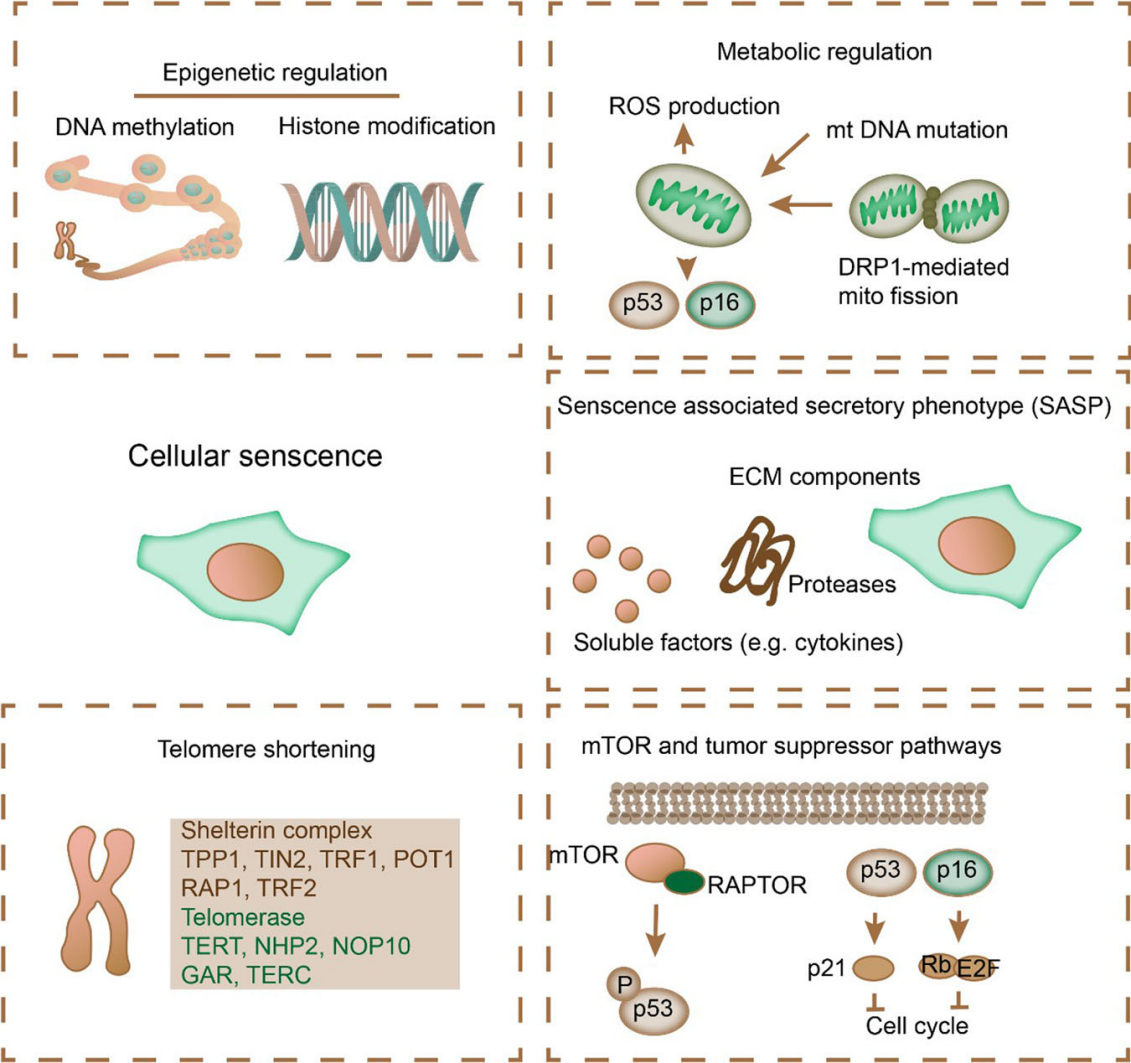
Molecular modulators of cellular senescence. Many factors can trigger cellular senescence, including epigenetic regulation, metabolic regulation, SASP, telomere shortening, mTOR, and tumor suppressor pathways.

Cell senescence can be triggered by various factors, such as epigenetic regulation, metabolic regulation, SASP, telomere shortening, mTOR, and tumor suppressor pathways (Fig. 3) [19]. Each factor contributes to the activation or inhibition of diverse signaling pathways. Meanwhile, alterations in these signaling pathways may affect related cellular processes, ultimately leading to cell senescence (Fig. 3). The molecular mechanism of senescence in diverse cell types may also differ. This section broadly reviews the molecular mechanisms in cell senescence.

#### Tumor suppressor proteins

Cellular senescence can be induced by activating the p53/p21 or p16/Rb pathways, resulting in cell cycle arrest (Fig. 3) [67]. In particular, p53 is associated with metabolism, DNA damage repair, autophagy, cell cycle, and apoptosis [68]. Various posttranslational modifications, such as acetylation, phosphorylation, and ubiquitination, modulate P53 activities. p21 is a cyclin-dependent kinase (CDK) inhibitor required for p53-dependent cell cycle arrest at the G1/S or G2/M phase. It binds to caspases to suppress cellular apoptosis, inducing cell senescence [69]. The p16/Rb pathway can be inhibited by p16, which binds to CDK4/6 to inhibit Rb phosphorylation and cell cycle arrest at the G1/S phase. These findings also demonstrate the role of the p16/Rb pathway in activating ROS and protein kinase C delta to maintain cell senescence [70].

#### Mechanistic target of rapamycin

Mechanistic target of rapamycin (mTOR), a serine/threonine kinase, modulates growth and metabolism in response to various external and internal signals [71, 72]. mTOR induces senescence via the activation of phosphoinositide 3-kinase (PI3K)/protein kinase B (AKT) and p53 signaling during oncogenic activation [73]. The two directions of cell fate, namely, senescence and quiescence, are determined by mTOR and p53; activation of both p53 and mTOR induces cellular senescence, whereas p53 activation and mTOR inhibition result in quiescence (Fig. 3) [73–75].

#### Epigenetic modulation of cell senescence

Epigenetic modifications, such as DNA methylation, histone modifications, chromatin remodeling, and noncoding RNAs, display a unique profile in senescent cells (Fig. 3). Senescent cells show a unique signature of DNA hypomethylation in late-replicative genes and certain promoter-proximal regions [62], which often manifests as cell cycle arrest. Sirtuins, a group of histone deacetylases, prevent senescent phenotypes in multiple cell types [37, 76]. The deacetylase activity of SIRT1 inhibits the transcription of SASP in cardiomyocytes, while in ECs, SIRT1 sustains EC functions by regulating endothelial nitric oxide synthase (eNOS) to alleviate oxidative damage [36, 77, 78]. miR-22 upregulation in aging hearts exacerbates senescence in cardiac fibroblasts [79]. Furthermore, miR-29 promoted aging by negatively regulating H4K20me3. The functions of other noncoding RNAs in cardiac aging have also been previously discussed [78]. LncRNAs promote cardiac regeneration and development by binding to ribonucleoproteins and miRNAs, forming the lncRNA-miRNA-target gene axis. For instance, lncRNA H19 induces cardiac senescence by suppressing miR-19 and activating the p53/p21 signaling pathway [80]. Moreover, ablation of lncRNA SNHG12 impaired DNA damage repair and vascular senescence, accelerating atherosclerosis [81].

#### Senescence-associated secretory phenotype

The SASP includes the secretion of soluble proinflammatory factors (cytokines, chemokines, and miRNAs), soluble cytokine receptors (TNF receptors), nonprotein soluble factors (nitric oxide), growth factors (EGF, VGEF, and NGF), and extracellular matrix macromolecules (fibronectin, collagens, and laminin) [4, 20]. Cytokines include IL-6 and IL-1 (Fig. 3). These findings reveal that the direct modulation of IL-6 is mediated by the DNA damage response (DDR) and its independence from p53, in addition to its influence on nearby cells harboring the IL-6 receptor on the surface [82]. Meanwhile, IL-1 is elevated during senescence, promoting the transcription of inflammatory cascades as a major consequence of the SASP phenotype [83]. The SASP mechanism is vital in transferring senescent signals from noncardiomyocytes to cardiomyocytes.

#### Circadian signals in senescence

Circadian rhythm dysregulation has been associated with reduced lifespan and immune cell senescence in mice [84]. For instance, mutation of the circadian gene per2 led to vascular senescence via the AKT pathway [85]. Misalignment in circadian rhythm may induce cardiovascular illnesses and cardiac aging since circadian rhythms regulate the rhythmicity of the heart [86]. Similarly, senescence in VSMCs may impair the transmission of circadian signals [87, 88].

### Mechanisms in cardiac senescence

Cellular senescence may lead to pathophysiological changes in cardiovascular system diseases, resulting in cardiac senescence. Cardiac senescence can be attributed to several factors, including metabolic dysfunction, telomere shortening, local communication, genetic predisposition, and epigenetic modification [23].

#### Metabolic dysregulation of cardiac senescence

The metabolic status of cardiomyocytes is unique compared to other cell types and is altered during development and physiological and pathological responses [89]. Metabolic profile changes significantly contribute to senescence in cardiomyocytes and heart functional decline, which have been documented (Fig. 3) [90]. Carnitine-palmitoyl transferase-1 (CPT1) was significantly reduced in the hearts of aging rats, possibly contributing to cardiac complications in pathological conditions [91]. Meanwhile, the loss of CPT1 during aging might exacerbate cardiac senescence and hypertrophy induced by pressure overload [92]. Additionally, peroxisome proliferator-activated receptor α (PPARα) and PGC-1α, which serve as regulators of fatty acid metabolism, decrease with aging [93]. Additionally, insulin signaling activation in cardiomyocytes promotes SASP and cardiomyocyte senescence [94], and p53 activation induces glycolysis to facilitate senescence (Fig. 3) [29]. Conversely, p53 suppression protects against senescence and diabetic cardiomyopathy [31]. Ketone bodies are minor substrates of oxidative metabolism in cardiomyocytes and are believed to be beneficial for cardiac function [95]. Aging causes elevated ketone bodies, including acetoacetate, b-hydroxybutyrate, and acetone. The energy source for cardiac senescence shifts to ketone bodies for oxidative ATP synthesis, which also minimizes oxidative and inflammatory damage in cardiomyocytes [96]. Cardiomyocyte-specific ketone body metabolism deficiency caused by succinyl-CoA:3-oxoacid CoA transferase (SCOT) deficiency results in mitochondrial stress and cardiac senescence [97]. The hexosamine biogenesis pathway is also involved in cardiac senescence [98]. Fructose 6-phosphate, an intermediate, can diverge into the hexosamine biogenesis pathway with the aid of glutamine fructose 6-phosphate amidotransferase (GFAT) [99]. O-linked-GlcNAc transferase uses uridine diphosphate-N-acetylglucosamine (UDP-GlcNAc) to initiate the O-GlcNAcylation (O-GlcNAc) of proteins [100]. Protein O-GlcNAcylation is a protective response during senescence that alleviates calcium overload, mitochondrial permeability transition pore opening, ER stress, and the heat shock response [101]. Notably, increased O-GlcNAc levels have been linked to both protective cardiac effects and ischemic injury [102].

Apart from the metabolic substrate pathway, the role of metabolic regulators in senescence, including AMP-activated protein kinase (AMPK), sirtuins, FOXOs, and mammalian target of rapamycin (mTOR), has also been discussed [103, 104]. AMPK modulates glucose and fatty acid metabolism and is inhibited in senescent myocardial tissues [105]. AMPK activation benefits mitochondrial dynamics, ER stress, and cardiomyocyte function while repressing cardiomyocyte senescence [106]. Sirtuins, including SIRT1, SIRT2, SIRT3, SIRT6, and SIRT7, are modulators of metabolism and senescence involved in cardiac senescence regulation [76]. SIRT2 regulates the metabolic and hypertrophic growth of cardiomyocytes in aging mice by targeting liver kinase B1 (LKB1)-AMPK signaling [107]. In summary, cardiomyocyte metabolism has been linked to cardiomyocyte senescence during the progression of aging and various disorders.

#### Mitochondrial dynamics and dysfunction

Dysregulation of mitochondrial fusion and fission processes causes mitochondrial elongation during senescence [108]. These findings have associated the senescent phenotype with decreased levels of the mitochondrial fission proteins FIS1 and dynamin-related protein 1 (DRP1) and increased levels of the mitochondrial fusion proteins mitofusins 1 and 2 (MFN1/2) and OPA1, leading to overlapping mitochondria [24, 109]. Mitochondrial fusion facilitates the normal function of mitochondria and the myocardium, while mitochondrial fission promotes the removal of damaged mitochondria by mitophagy [24]. The imbalance between fusion and fission leads to the retention of damaged mitochondria, subsequently accumulating oxidized proteins and aggravating senescent phenotypes. The accumulation of dysfunctional mitochondria induces oxidative damage to lipids and mitochondrial DNA [24]. Metabolic disturbance associated with dysfunctional mitochondria generally leads to direct cardiac senescence.

#### Sirtuins

The deacetylase sirtuins are widely acknowledged as NAD-dependent enzymes closely related to caloric restriction (CR) and longevity [110]. The sirtuin family comprises seven members (SIRT1-7), of which SIRT1-3, SIRT6, and SIRT7 exhibit cardioprotective activities [111]. The study of SIRT1 is extensive due to its effects on longevity and cardioprotection [77]. Cardiac-specific depletion of SIRT1 manifested aging-related metabolic profiles during ischemia/reperfusion stimulation [112]. SIRT2 protects against angiotensin II-induced cardiac hypertrophy by deacetylating STK11 and activating AMPK signaling [107]. SIRT3, SIRT4, and SIRT5 have been studied in the context of mitochondrial metabolic processes, oxidative stress, and mitochondrial dynamics, which are closely associated with cardiovascular diseases and aging [113]. Research has also revealed the anti-aging effects of SIRT6 that might contribute to telomere preservation, DNA repair, genomic stability, and glucose metabolism [46]. SIRT6 or SIRT7 deficiency might lead to premature aging features, while SNPs in SIRT5 and SIRT7 have been linked to longevity and CR [37]. These findings indicate the engagement of the sirtuin family in a complex regulatory network that mediates cardiac homeostasis, aging, and metabolism and could potentially act as an anti-aging target. Natural dietary supplements that modulate the activity of sirtuins are expected to provide beneficial effects in impeding cellular aging and improving metabolism. The intake of Sirtfood (food rich in sirtuin activators) has received much attention for its role in enhancing metabolism and lifespan [114].

#### Local microenvironment of cardiac senescence

The local microenvironment can be remodeled by aging and age-related pathological conditions [115]. In pathological conditions, different components interact, leading to heart dysregulation. Noncardiomyocytes influence cardiomyocytes and contribute to cardiac aging by releasing signals. (Fig. 1) [116].

#### Interaction of ECs and cardiomyocytes

Metabolic changes in ECs during senescence were previously reported (Fig. 1) [117]. ECs function as vascular barriers and secrete cells that produce paracrine factors to regulate the local microenvironment [118]. The secretion of endothelial-specific paracrine and inflammatory factors modulates cardiomyocyte senescence and cardiac functions [119]. During embryonic and postnatal stages, ECs produce parathyroid hormone-related peptide (PTHRP), nitric oxide (NO), endothelin 1 (ET-1), platelet-derived growth factor (PDGF)-B, angiotensin II (Ang II), prostacyclin (PGI2), prostaglandin E2 (PGE2), and neuregulin-1 (NRG1) to facilitate the proliferation and maturation of cardiomyocytes [120]. ECs also secrete apelin, proinflammatory factors (TGFβ and IL6), and eicosanoids in addition to Ang II, NRG1, and ET-1 following metabolic stress, contributing to cardiomyocyte senescence (Fig. 1) [121, 122]. The role of ET-1 in the progression of cardiomyocyte senescence and cardiovascular diseases was previously described [123]. Meanwhile, the elevation of Ang II and ET-1 induced age-related cardiac hypertrophy and fibrosis [124]. Conversely, suppressing endothelin receptor A (ETAR) protects the heart from cardiac hypertrophy and contractile incapability caused by aging or high fat via autophagy [125]. Cardiac ECs can also produce exosomes, including microRNAs (miRNA-126-3p and miRNA-5p) [126] or extracellular vehicles, to modulate cardiomyocyte functions (Fig. 1) [127]. A study reported that exosomal Mst1 suppresses autophagy, induces apoptosis in cardiomyocytes, and inhibits glucose metabolism under diabetic conditions [128]. Liver kinase B1 (LKB1) can modulate cell polarity and energy homeostasis by activating AMPK [129]. Depletion of LKB1 in ECs leads to EC dysfunction, subsequently triggering hypertension and cardiac hypertrophy [130]. LKB1-AMPK signaling may also modulate cardiomyocyte senescence via the paracrine pathway [131]. These findings demonstrate the roles of ECs in modulating the proliferation, differentiation, and senescence of cardiomyocytes by secreting angiocrine and proinflammatory cytokines [132].

#### Roles of fibroblasts in cardiomyocyte senescence

Fibroblasts are one of the primary cardiac tissues vital for modulating cardiac function via interactions with cardiomyocytes [133]. In addition to their role in cardiac development and homeostasis, fibroblasts contribute to cardiac remodeling and diseases [134]. Similarly, the fibroblast-cardiomyocyte interaction is mediated by paracrine secretion, including Ang II, cardiotrophin 1, fibroblast growth factor (FGF), IL-6, insulin-like growth factor 1 (IGF1), TGFβ, and TNFα (Fig. 1) [135]. Fibroblasts also produce IL-33 to inhibit cardiomyocyte senescence triggered by hypoxia and hypertrophic damage [136]. IL-11 secreted by fibroblasts is involved in cardiac dysfunction and hypertrophy [137]. ATPase 4 signaling in fibroblasts regulates cardiac hypertrophy by facilitating the expression and secretion of frizzled-related protein 2 (sFRP2) [138]. The role of exosome-derived miRNAs in modulating cardiomyocyte function has also been discussed [139]. Exosome-derived miR-21-3p reportedly triggers cardiac hypertrophy via sorbin and SH3 domain-containing protein 2 (SORBS2) and PDZ and LIM domain 5 (PDLIM5) [140]. Cardiac fibroblasts modulate cardiomyocyte senescence through paracrine secretion and ECM remodeling [141]. Integrins and matrix metalloproteinases (MMPs) produced by fibroblasts promote vital adhesive and signaling functions by interacting with ECM and actin, which are key components in paracrine signaling, ECM homeostasis, cardiomyocyte senescence, and other pathological alterations (Fig. 1) [142]. Furthermore, fibronectin plays a vital role in the buildup of cardiac myofibroblasts after cardiac damage and remodeling [143].

The metabolic state of fibroblasts also influences their function during cardiac aging and remodeling [144]. APPL1-AMPK signaling is activated by adiponectin, which further promotes cell migration, MMP activation, and collagen remodeling in cardiac fibroblasts [145]. AMPK activation induces fibroblast accumulation in infarcted areas [146]. FOXO3A prevents oxidative stress in cardiac fibroblasts by mediating peroxiredoxin III levels [147]. Therefore, metabolic regulators in cardiac fibroblasts can mediate the function and senescence of cardiomyocytes.

#### Immune cells in cardiomyocyte senescence

Immune cells, including macrophages, mast cells, and T cells, modulate tissue homeostasis and pathogenesis by regulating the inflammatory response and cardiomyocyte senescence in cardiac tissues (Fig. 1) [4]. A previous study reported the role of macrophages in the pathological process of cardiac tissues [148]. Macrophages participate in cardiac tissue development, regeneration, and pathological remodeling [148]. Meanwhile, the macrophage-specific deletion of connexin 43 resulted in cardiomyocyte senescence and impaired electrical activity [149]. Activation of the NLRP3 inflammasome in cardiac macrophages directly stimulates the production of IL-1β, resulting in cardiomyocyte senescence due to prolonged action potential in cardiomyocytes in diabetic mice [150]. Similar macrophage roles have been reported in atrial fibrillation. Depletion of hepcidin, an iron regulator, led to chemokine receptor 2 (CCR2) inflammatory macrophage accumulation, accelerating the phosphorylation of signal transducer and activator of transcription factor-3 (STAT3) and promoting cardiomyocyte renewal [151]. These findings indicated a close association between macrophages, cardiomyocyte renewal and senescence. However, the particular subtypes of macrophages involved in cardiomyocyte renewal and senescence remain unknown, as do the paracrine factors in macrophages.

Despite its established role in modulating adaptive immunity, the role of T cells in cardiac diseases remains unclear. T cells are the primary producers of IL-17A, which promotes cardiomyocyte apoptosis following cardiac ischemia/reperfusion [152]. T-cell activation inhibition prevents cardiomyocyte apoptosis and hypertrophic growth in response to pressure overload and heart failure [153]. Meanwhile, the adoptive transfer of CD4^+^CD25^+^ regulatory T (Treg) cells alleviates Ang II-induced cardiac injury and fibrosis [154]. The paracrine secretion of cystatin F, TNF superfamily member 11 (TNFSF11), IL-33, fibrinogen-like protein 2 (FGL-2), matrilin-2, and IGF-2 induces proliferation and represses senescence in cardiomyocytes [155]. In addition to macrophages and T cells, mast cells are responsible for inflammation and tissue remodeling [156]. Depletion of mast cells reduced cardiomyocyte contractility and impaired post-ischemia cardiac function [157]. Furthermore, mast cells produce chymase to exacerbate cardiomyocyte hypertrophy and senescence [158]. These findings suggest the critical role of mast cells in modulating cardiomyocyte function and senescence via paracrine pathways. Activating immune cells also involve the role of metabolic regulators [159], including metabolic reprogramming, which facilitates macrophage polarization [160]. Typically, type I macrophages (M1) rely on glycolysis for energy supply [160], whereas type II macrophages (M2) use oxidative metabolism for energy supply upon wound healing and tissue repair [161]. AMPK alleviates the inflammatory response and is associated with aging-related cardiovascular diseases [162].

#### Genetics of cardiac senescence

Currently, there is no conclusive evidence suggesting the causation of genetics in human lifespan and cardiac aging. Research has estimated that genetic factors account for 1/4 of the diversity in the human lifespan, with the remainder influenced by the complex interaction of genes, environment, lifestyle, and epigenetics [163]. Elucidating the metabolic pathways that affect the human lifespan has led to the discovery of genes that potentially influence lifespan and aging. Genome-wide association studies (GWAS) have shed light on preventing and treating cardiac aging and diseases by identifying new genetic loci and pathways involved in late-onset cardiovascular diseases [164]. Proximate good genes potentially predispose people to longevity, while genetic mutations such as progeria or Werner syndrome, lead to premature aging. Mutations in the LMNA gene contribute to DNA damage and premature death in progeria [165]. Research has also shown that people who exhibit typical aging characteristics at an early age are prone to cardiovascular disease [166]. Werner syndrome, another accelerated aging condition, is caused by mutations in the WRN gene, which encodes a helicase enzyme involved in double-strand DNA break repair and genome stability [167]. These findings have linked APOE and FOXO3 to human longevity [168]. Four genetic variants, ABO, APOE-TOMM40, CDKN2B-ANRIL, and SH2B3-ATXN2, have also been associated with longevity [169]. Another study of elderly individuals revealed that 5q33.3 is a novel locus related to low blood pressure in middle age and a lowered risk of cardiovascular diseases [170]. A comparison of human lifespan in China, Europe, and the United States revealed numerous overlapping SNPs, identified APOE and 5q33.3 as loci related to longevity, and suggested four pathways (carbohydrate metabolism, immune response, MAPK signaling, and calcium signaling) [171]. Evidence has demonstrated that genetic and environmental risk factors might influence lifespan and that no one gene can ensure longevity. Smoking, diabetes, and coronary artery diseases have been shown to correlate negatively with longevity, while education and openness to experience are positively correlated with longevity [172].

#### Epigenetic effects on cardiac senescence

Epigenetic modification alters gene function independent of changes in gene sequence and depends on DNA methylation, heterochromatin or chromatin remodeling, histone modifications, and noncoding RNAs [173, 174]. Epigenetics can be stable, heritable, rapid and reversible in response to stimuli and environmental alterations [175]. During aging, these alterations affect chromatin accessibility, gene expression, and genome stability [176]. Understanding epigenetics during aging helps discover potential biomarkers for aging to treat aging-associated disorders.

#### DNA methylation

DNA methylation has been extensively discussed concerning genetic regulation, aging, and cardiac diseases [175]. DNA methyltransferases facilitate DNA methylation at the cytosine ring in CpG islands. The methylation sites near gene transcription initiation sites typically block the initiation of gene transcription by providing steric hindrance to transcription factors or promoting repressive chromatin modifications [177]. DNA methylation generally accumulates (hypermethylation) at sites and decreases (hypomethylation) during replicative senescence [178]. Inhibition of DNA methylation in cardiac diseases leads to atherosclerosis [179]. Research has revealed the association between homocysteine accumulation (a precursor of methionine) and coronary artery disease, indicating the correlation between plasma homocysteine levels and global DNA methylation levels in patients with coronary artery disease [180]. The global variance of DNA methylation in HGPS or Werner syndrome is independent of LMNA or WRN mutations, indicating the role of DNA methylation in aging syndrome [181].

A study showed similar DNA methylation patterns in different species during aging, a condition known as methylation drift or epigenetic drift [182]. Research has also identified potential biomarkers for human lifespan produced by enhanced DNA methylation drift in cells and tissues during aging [183]. Notably, CR retards DNA methylation drift and facilitates a younger epigenetic profile in mice and monkeys, depicting the dynamic interaction of external and intrinsic factors [184].

#### Histones

Histones are core proteins wrapped by eukaryotic DNA and packaged into nucleosomes [175]. Histone depletion is recognized as a typical feature of cardiac aging, showing reduced histone expression, maturation, or deposition [185]. Histone depletion is caused by DNA damage during replicative senescence. Research has shown that histone loss and reduced histone occupancy increase with age in all species, leading to age-associated chromatin disorganization and genomic instability [186]. Under some conditions, these alterations are associated with myocardial regenerative processes. For instance, the substitution of histone 3 (H3) with histone variant H3.3 in proliferating cardiomyocytes resulted in faulty DNA repair and SASP phenotypes [187]. Meanwhile, H3 replacement with another histone variant, histone H3-like centromeric protein A (CENPA), is crucial for kinetochore formation and function during cell division [188]. The CENPA level decreases significantly with aging in human pancreatic islet cells and mouse CPCs [189]. CENPA depletion triggers premature aging in mouse CPCs accompanied by exacerbated cell death [190]. Research has also shown that histone overexpression can expand the lifespan of yeast [191]. Histone variants, such as H3.3 and CENPA, can alter histone accessibility and may offer an approach for intervening in myocardial aging processes. In addition to histone levels and components, histone post-translational modifications can also modulate chromatin structure and cell senescence [192]. Histone modifications include acetylation and methylation, which occur on the residues of the histone amino-terminal tail region. Acetylation is typically an activating modification that changes with aging [193]. Acetylation or methylation of histone lysine sites is also associated with longevity. Histone acetyltransferases loosen the nucleosome by transferring the acetyl group to histones to facilitate gene transcription. Conversely, histone acetyltransferases remove the acetyl group, promoting histone-DNA interactions and increasing nucleosome compaction [174]. Histone deacetylases are classified into four groups, and their activities depend on either zinc or NAD^+^. The role of histone methylation in aging relies on its transcriptional regulation, which can regulate autophagy, cellular senescence, DNA damage, and environmental stress [194]. Enzymes that directly modulate histone methylation and acetylation serve as promising targets for mitigating aging processes.

#### Noncoding RNAs

Noncoding RNAs, including microRNAs (miRNAs) and long noncoding RNAs (lncRNAs), play an important role in cardiac senescence and CVD progression [195]. For instance, upregulation of miRNA-22 in aging hearts induced aggravated cardiac fibroblast senescence and migratory activity [79]. Moreover, miR-34a plays an important role in aging-associated CVD. Previous research revealed that miR-34a activation after cardiac injury induced elevated apoptosis in cardiomyocytes. MiR-34a was observed to be upregulated with age in mouse cardiomyocytes. LncRNAs have emerged as critical modulators of cardiac aging. Metastasis-associated lung adenocarcinoma transcript 1 (MALAT1) is directly linked to miR-34a. MALAT1 downregulates the level of miR-34a. As revealed above, miR-34a is upregulated with age, which may be due to the downregulation of MALAT1. LncRNA H19 is closely related to aging, which negatively modulates cell proliferation. Additionally, H19 is associated with the regulation of cell senescence. Therefore, H19 might be a promising target in senescence-related diseases. The H19 locus is hypomethylated in multipotent germline stem cells, accompanied by a high level of telomerase [196]. However, the detailed roles of H19 in biological aging are currently inadequate. While lncRNAs are implicated in aging regulation, whether lncRNAs can act as potent therapeutics in antiaging remains to be addressed [197].

#### Telomeres on cardiac senescence

Telomeres, referring to the protective and stabilizing ends in eukaryotic chromosomes, are made up of short DNA repeats, telomerase RNA component (TERC), and TERT [198]. Some connecting proteins are collectively designated shelterins, which are involved in telomere maintenance and genomic stability. In response to cell division, inflammation, or oxidative stress, telomeres are shortened and ultimately reach a minimum length, which might contribute to cell cycle arrest, chromosome damage, or senescence [199]. Therefore, the length of telomeres is recognized as a marker for cell senescence. Research has associated telomere length with cellular division capacity, indicating tissue and organ age [200]. Telomere length, TERT expression, and telomerase activity reportedly decreased significantly in postnatal cardiomyocytes. Telomere shortening is primarily caused by inflammation and oxidative stress in cardiomyocytes [201]. Meanwhile, findings suggest that telomere length in noncardiomyocytes is a marker for cardiac age and diseases [202]. Blood can also be a perfect source for identifying telomere length, suggesting its use as a diagnostic tool for human aging.

#### Damaged mitochondria and autophagy in cardiac senescence

Autophagy is found to decline in efficiency as aging progresses, especially in the heart, liver, and nervous system [203]. Due to the accumulation of oxidative damaged components and ROS, a corresponding enhancement in autophagy is believed to be beneficial. In fact, inhibition of autophagy can exacerbate ROS production. Cardiac inhibition of the autophagy gene Atg5 leads to left ventricular hypertrophy, damaged sarcomere structure, and dysfunctional mitochondria accumulation [204]. Apart from damaged mitochondria accumulation, aged myocardium in rodents also showed a nondegradable yellow-brown pigment called lipofuscin, which is induced by ROS-induced modification of proteins and lipids within lysosomes. Lysosomes with lipofuscin consume a large majority of newly produced hydrolases that cannot degrade lipofuscin [205]. Therefore, only a small proportion of lysosomal enzymes can be used for autophagic degradation, eventually leading to an overburdened autophagy-lysosomal system and decreased degradative capacity. Cardiac-specific deletion of lysosomal DNase II in mice under pressure overload resulted in an activated inflammatory response in myocytes, which is a hallmark of cardiac senescence [206]. Abnormalities in mitochondrial structure and function are implicated in a number of pathological conditions in the cardiovascular system. Autophagy induction in these conditions is regarded as a rescue process by clearing damaged mitochondria.

### Promising therapy for cardiac senescence and future directions

**Fig. 4:**
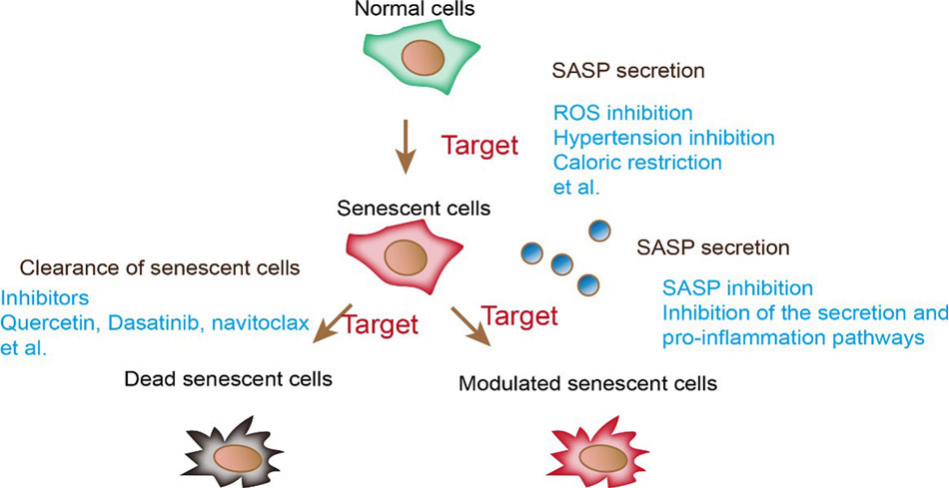
Promising therapies for targeting cellular senescence in aging hearts. Strategies targeting senescence include clearance of senescent cells, modulation of senescent cells, and regulation of SASP secretion. Quercetin, dasatinib, and navitoclax have been proven to remove senescent cells. ROS inhibition, hypertension inhibition, and CR prevent SASP secretion.

Some chemotherapies, including anthracyclines and doxorubicin, can induce cardiac senescence in multiple cell types and late heart failure. Doxorubicin damaged mtDNA in rat neonatal cardiomyocytes and elevated p16 levels and SA-β-Gal activity (Fig. 4) [207]. Doxorubicin also induces VSMC senescence and reduces SIRT1 levels and AMPK activation, creating a proinflammatory microenvironment [208]. A study demonstrated that prednisolone could prevent doxorubicin-induced VSMC senescence [209]. LncRNA-MALAT1 protects against doxorubicin-induced senescence and mitochondrial dysfunction by suppressing autophagy-regulating protease 4a (ATG4a) [210]. Therapies targeting chemotherapy-induced cellular senescence may provide an alternative to anthracycline toxicity.

Typical signatures of senescent cells, such as increased glycolysis, DDR, and SASP, make them a potential target for removing senescent cells (senolytics) or alleviating senescent features (senostatics) (Fig. 4). Dasatinib and quercetin remain the first senolytic drugs identified that promote apoptosis in senescent cells (Fig. 4) [211]. Dasatinib, a tyrosine kinase inhibitor, promotes apoptosis by interrupting the binding of ephrin ligands with ephrin receptors in senescent cells. Quercetin, a flavonol, suppresses PI3K and mTOR signaling and activates SIRT1. Quercetin also alleviates lipid deposition in the aortas of *ApoE*-deficient mice. Coadministration of quercetin and dasatinib effectively reduced the level of p16 in senescent cells following ionizing radiation exposure and improved left ventricular systolic function in aged mice [211]. Furthermore, cotreatment with quercetin and dasatinib improved senescent phenotypes and vasomotor function in a hypercholesterolemia model.

Navitoclax can induce apoptosis in senescent lung fibroblasts but not preadipocytes [212]. Navitoclax facilitated the removal of senescent cells in the myocardium after MI and ameliorated survival and fibrosis in mice (Fig. 4). Navitoclax also improved left ventricular systolic function, cardiac fibrosis, hypertrophy, and conduction velocity in mice with angiotensin II (Ang II)-induced heart failure [213].

Cardiac glycosides, a class of compounds originating from foxglove plants, include digoxin, ouabain, and proscillaridin. Cardiac glycosides have been associated with the inhibition of the Naþ/Kþ ATPase pump, which pumps sodium out of the cell and potassium into the cell against concentration gradients using ATP, disrupting the electrochemical gradient. Senescent cells display a more polarized state than normal cells, making them more susceptible to the effects of cardiac glycosides. Administration of digoxin can effectively remove senescent pulmonary fibroblasts in a pulmonary fibrosis mouse model [214].

CR is a robust anti-aging intervention. A prominent mechanism by which CR extends lifespan can be attributed to its modulation of the autophagic response [215]. The inhibition of autophagy in lower organisms blocks the anti-aging effect of CR. CR induces macroautophagy via different pathways: the insulin-like growth factor-1/insulin signaling pathway, the sirtuin pathway, the AMPK pathway, and the mTOR pathway, which are intimately interconnected and play vital roles in mediating different aspects of this response. As noted in a study, lifelong 40% CR elevated the levels of Atg7, Atg9, and LC3-II in the hearts of old rats [216].

Statins, such as simvastatin, are common interventions for high cholesterol levels and atherosclerotic complications in older adults. They can effectively inhibit cholesterol synthesis by suppressing β-hydroxy β-methylglutaryl-coenzyme A (HMG-CoA) reductase, the rate-limiting enzyme in cholesterol synthesis. Statins inhibit senescent growth arrest and reduce senescent markers in endothelial progenitor cells [64]. Statins also suppress the proinflammatory SASP in senescent fibroblasts, which is independent of the cholesterol effect.

Resveratrol treatment alters senescent gene expression profiles in old mice [217]. Moreover, resveratrol rescued the survival rate and prevented cardiac pathology in mice fed a high-calorie diet. Resveratrol treatment in rodents suppresses apoptosis in cardiomyocytes, prevents ischemia/reperfusion injury in myocardium, and reduces inflammation. The beneficial effects of resveratrol on cardiac senescence can partially be attributed to its effect in activating SIRT1, enhancing endothelial NO signaling, and activating Nrf2 in endothelial cells.

As mentioned, the metabolic modulators AMPK, mTOR, and PI3K are implicated in senescent cells. These findings also suggest a critical role of inhibitors in metabolic pathways regulating cell senescence. Rapamycin has been shown to remove progeria via improved autophagy and reduce senescence in patients with Hutchinson-Gilford progeria syndrome, as inhibition of the mTOR pathway can lengthen life expectancy in mice [218]. Celastrol reduces intracellular ROS and prevents Ang II-induced senescence by inhibiting mTOR signaling and enhancing autophagy in VSMCs [219]. Rapamycin has been shown to remove progeria via improved autophagy and reduce senescence in patients with Hutchinson-Gilford progeria syndrome, as inhibition of the mTOR pathway can lengthen life expectancy in mice [220]. Metformin protects against EC senescence and suppresses atherosclerotic plaque formation in *ApoE*^-/-^ mice. Moreover, metformin treatment can restore autophagy and disrupt the p62-RIP1-RIP3 complexes, effectively reducing I/R-induced necroptosis in aging hearts [221].

SASP includes multiple factors that vary with the microenvironment, and the inhibition of SASP can block the corresponding inflammatory response and alleviate cell senescence. Melatonin was revealed to inhibit SASP in human primary fetal lung fibroblasts and human embryonic fibroblasts mediated by poly (ADP-ribose) polymerase-1 (PARP-1) and CREB-binding protein, thus inhibiting acetylation of H2BK120 and restraining elevation of SASP genes [222]. Calcitonin gene-related peptides can also reduce SASP and prevent senescence in cardiac fibroblasts. Since many pathways can activate the SASP, which is important in diverse physiological processes, such as tumor surveillance and the immune system, the disturbance of the SASP without interrupting these processes remains challenging.

Cardiac progenitor cells (CPCs) are incapable of proliferating during aging due to telomere shortening, dysregulated cell division, and activation of senescence markers, leading to complete cardiac aging [223]. Telomerase-deficient hematopoietic stem cell accumulation during serial transplantation leads to decreased replicative lifespan [224]. Similarly, bone marrow-derived mononuclear cells from ischemic cardiac disease manifested several senescent signatures, including telomere shortening, myeloid differentiation capacity alleviation, and increased p21 and p16INK4A expression [225]. The powerful regenerative repair ability and intervention effect mediated by stem cells broaden the current clinical treatment direction and means for age-related cardiovascular diseases and show unique advantages and application prospects in the intervention of cardiac aging. An extreme approach to restoring youth in aged mice and humans involves reprogramming cells into induced pluripotent stem cells (iPSCs). However, the application of iPSCs for the treatment of heart diseases can be challenging [226]. Applying iPSCs in antiaging treatments seems promising [227]. To summarize, these findings suggest that restoring functional impairment in senescent stem cells remains a significant obstacle in the pursuit of optimal and effective personalized regenerative therapy.

#### Personalized strategy

Precision medicine is dedicated to providing personalized treatment for individual patients with specialized disease phenotypes and molecular mechanisms. The molecular mechanism of individual cardiac senescence can be used as a target to prevent diseases. Nuclear β-dystroglycan (β-DG) is associated with the maintenance of nuclear architecture and function. Loss of β-DG drives cellular senescence via defective mitosis-mediated genomic instability. Precision medicine has also developed a method for correcting a frameshift mutation in the dystrophin gene in Duchenne muscular dystrophy (DMD) patients. A broad understanding of the epigenetic, proteomic, and metabolomic mechanisms of diseases enables a comprehensive interpretation of disease mechanisms and potential exploration of effective disease targets.

For patients with heart failure reduced ejection fraction (HFrEF) and related left ventricular (LV) hypertrophy with unidentified underlying mechanisms, precision-based therapies include genetic evaluation for hypertrophic cardiomyopathy, Fabry disease, Noonan syndrome, or other genetic alterations of LV hypertrophy. Meanwhile, evaluation for possible cardiac amyloidosis by imaging modalities is also necessary [228]. In this case, the identification of a specific cause of heart failure with preserved ejection fraction (HFpEF) with LV hypertrophy can promote targeted therapies. In patients with nonischemic cardiomyopathy and HFrEF, evaluation of genetic etiologies can facilitate specific management (i.e., LMNA-associated cardiomyopathies) and clinical screening. Personalized antiplatelet therapy was noninferior to ticagrelor with respect to all-cause mortality and readmissions in a case‒control study of acute coronary syndrome patients genotyped for CYP2C19 [229]. It also led to more appropriate use of both clopidogrel and ticagrelor with resultant cost savings. Dalcetrapib is a drug designed to elevate the level of high-density lipoproteins by inhibiting cholesteryl ester transfer protein (CETP). Although dalcetrapib is disappointing in clinical trials, a retrospective study indicated that dalcetrapib may be more beneficial in individuals with a specific polymorphism in the adenylate cyclase gene 9. Relevant clinical trials are underway to validate this hypothesis directly [230].

A cell transplantation strategy has been proposed to replace dysfunctional cells with differentiated human cells originating from human stem cells. Cells in the heart normally lose the ability to proliferate; thus, it would be interesting to transplant stem cell-derived cardiomyocytes into the heart during heart failure. Interestingly, transplantation of young bone marrow Sca-1+ stem cells, rejuvenates the aged heart and improves function after injury. Nonetheless, the interaction of immune cells and surrounding cardiac cells should be taken into consideration when developing cell-based approaches for cardiac rejuvenation.

Although precision medicine is a clear mainstream therapy, it is still debated whether precision medicine can have a global effect on the prevention and treatment of cardiovascular disease and whether it is only beneficial for a small proportion of patients. This skepticism has arisen, partially due to advances in this field and challenges in maintaining the physician‒patient relationship. Meanwhile, precision medicine relies on large data acquisition and methods to ensure data accuracy, computational capacity, security and privacy of data, renewal of data, and continuous refinement of analytic methods. Moreover, the successful implementation of precision medicine requires education, affordability, and public acceptance of the strategy.

## Conclusions

Cardiac senescence is gradually recognized as a potential cause of disease phenotypes within the cardiovascular system. A comprehensive understanding of the way that each cell type contributes to cardiac and main signaling pathways in cardiac senescence would help us to develop effective and specialized strategies to protect and slow aging and related diseases in the heart. Current strategies aim to remove or suppress senescent cells. Future advances in epigenetic, metabolomic or proteomic mechanisms of cardiac senescence may offer personalized therapeutics to patients with aging-related diseases.

## Data Availability

All data generated or analyzed in this study are available from the corresponding author upon reasonable request.

## Materials availability

All data generated or analyzed in this study are available from the corresponding author upon reasonable request.
